# Effectiveness of a mHealth intervention on hypertension control in a low-resource rural setting: A randomized clinical trial

**DOI:** 10.3389/fpubh.2023.1049396

**Published:** 2023-03-01

**Authors:** Zhang Yuting, Tan Xiaodong, Wang Qun

**Affiliations:** ^1^Health Science Centre, Shenzhen University, Shenzhen, China; ^2^School of Public Health, Wuhan University, Wuhan, China

**Keywords:** mHealth, hypertension, low-resource rural settings, randomized clinical trial, behavior intervention

## Abstract

**Background:**

Despite the increasing popularity of mHealth, little evidence indicates that they can improve health outcomes. Mobile health interventions (mHealth) have been shown as an attractive approach for health-care systems with limited resources. To determine whether mHealth would reduce blood pressure, promote weight loss, and improve hypertension compliance, self-efficacy and life quality in individuals with hypertension living in low-resource rural settings in Hubei, China.

**Methods:**

In this parallel-group, randomized controlled trial, we recruited individuals from health-care centers, home visits, and community centers in low-resource rural settings in Hubei, China. Of 200 participants who were screened, 148 completed consent, met inclusion criteria, and were randomly assigned in a ratio of 1:1 to control or intervention. Intervention group participants were instructed to use the Monitoring Wearable Device and download a Smartphone Application, which includes reminder alerts, adherence reports, medical instruction and optional family support. Changes in the index of Cardiovascular health risk factors from baseline to end of follow-up. Secondary outcomes were change in hypertension compliance, self-efficacy and life quality at 12 weeks.

**Results:**

Participants (*n* = 134; 66 in the intervention group and 68 controls) had a mean age of 61.73 years, 61.94% were male. After 12 weeks, the mean (SD) systolic blood pressure decreased by 8.52 (19.73) mm Hg in the intervention group and by 1.25 (12.47) mm Hg in the control group (between-group difference, −7.265 mm Hg; 95% CI, −12.89 to −1.64 mm Hg; *P* = 0.012), While, there was no difference in the change in diastolic blood pressure between the two groups (between-group difference, −0.41 mm Hg; 95% CI, −3.56 to 2.74 mm Hg; *P* = 0.797). After 12 weeks of follow-up, the mean (SD) hypertension compliance increased by 7.35 (7.31) in the intervention group and by 3.01 (4.92) in the control group (between-group difference, 4.334; 95% CI, 2.21 to −6.46; *P* < 0.01), the mean (SD) hypertension compliance increased by 12.89 (11.95) in the intervention group and by 5.43 (10.54) in the control group (between-group difference, 7.47; 95% CI, 3.62 to 11.31; *P* < 0.01), the mean (SD) physical health increased by 12.21 (10.77) in the intervention group and by 1.54 (7.18) in the control group (between-group difference, 10.66; 95% CI, 7.54–13.78; *P* < 0.01), the mean (SD) mental health increased by 13.17 (9.25) in the intervention group and by 2.55 (5.99) in the control group (between-group difference, 10.93; 95% CI, 7.74 to 14.12; *P* < 0.01).

**Conclusions:**

Among participants with uncontrolled hypertension, individuals randomized to use a monitoring wearable device with a smartphone application had a significant improvement in self-reported hypertension compliance, self-efficacy, life quality, weight loss and diastolic blood pressure, but no change in systolic blood pressure compared with controls.

## Introduction

Hypertension is the most common chronic condition for cardiovascular and cerebrovascular events worldwide, affecting 32.6% of US adults, and has an estimated annual medical expenses exceeding $50 billion (1, 2). Worldwide, 422.7 million people diagnosis with cardiovascular disease (3), and causes 16.7 million deaths each year, 80% of which occur in low-income and middle-income countries (4). According to a recent investigation, in rural China, the control and control under-treatment rate of hypertension were only 8.6 and 19.8%, respectively (5). Decades of research have shown that even the modest reductions in blood pressure (BP) would reduce the premature mortality and the risk of associated morbidity (6). However, despite the widespread availability of well-tolerated, effective, and inexpensive drugs, approximately half of treated patients do not have well-controlled BP (7). Lack of patient engagement, poor medication adherence, and therapeutic inertia are major contributors to patients not reaching their recommended BP levels (8).

Many types of intervention methods have been conducted to improve therapeutic targets and BP control. Systematic reviews summarizing more than 3 decades of research advocate for specific lifestyle modifications in populations with high risk of cardiovascular disease (9, 10). In addition, improvement of patients' self-management, nurses and pharmacists have also been proved to be effective in hypertension control in team-based care (11, 12). However, in favor of lifestyle modifications for the reduction of cardiovascular disease risk is mostly restricted to trials done in high-income countries (13). Few trials have been done in low-income and middle-income countries, despite robust evidence supporting their effectiveness (14).

With the rapid rise and popularity in mobile phone use, mobile health (mHealth) could become a potential way to address several health-care system constraints in low and middle income countries, such as limited medical resources, overburdened health-care workforce, and an increasing prevalence of chronic diseases (4). In view of all these constraints, it is very challenging to extend the health care to difficult-to-reach populations. Strategies that depend on offering education, providing reminders for medication taking and refilling, or facilitating social interactions have been shown to increase physical activity, promote weight loss, encourage behavior change and improve patient-provider communication (15–17). In a systematic review, use of mobile apps and SMS messaging was found to improve physical health and reduce stress, anxiety, and depression, and the review showed using mobile apps and SMS text messaging as promising mHealth interventions (18). However, a systematic review (19) showed that m-health interventions had a positive effect on chronic diseases and also highlighted the need for more rigorous research in developing countries. Since, only 9 trials from low and middle-income countries were included in the analysis, and only 1 of them conducted in China.

In our research, we aimed to investigate whether mHealth including wearable monitoring device support home-based self-monitoring weekly counseling phone calls and advice for lifestyle modification could reduce BP, promote weight loss, and improve hypertension Compliance, self-efficacy and quality of life in adults with hypertension living in low-resource rural settings in China.

## Methods

### Study design

The Self-Monitoring Intervention Programme for Hypertension Control was a randomized trial conducted among 6 primary care centers within a remote mountainous districts of Hubei province, China. Details of the Program's study design and organizations have been published elsewhere (20).

All the selected primary care centers were located in a poor rural area and provided free medication and health care to hypertensive patients. Three centers were assigned to the mobile health intervention and the other 3 centers to usual health care. All participants were included consecutively to avoid selection bias. Given the nature of the behavioral intervention, no action was taken to balance the recruitment for individuals that refused consent.

### Conceptual framework

**Figure 1:**
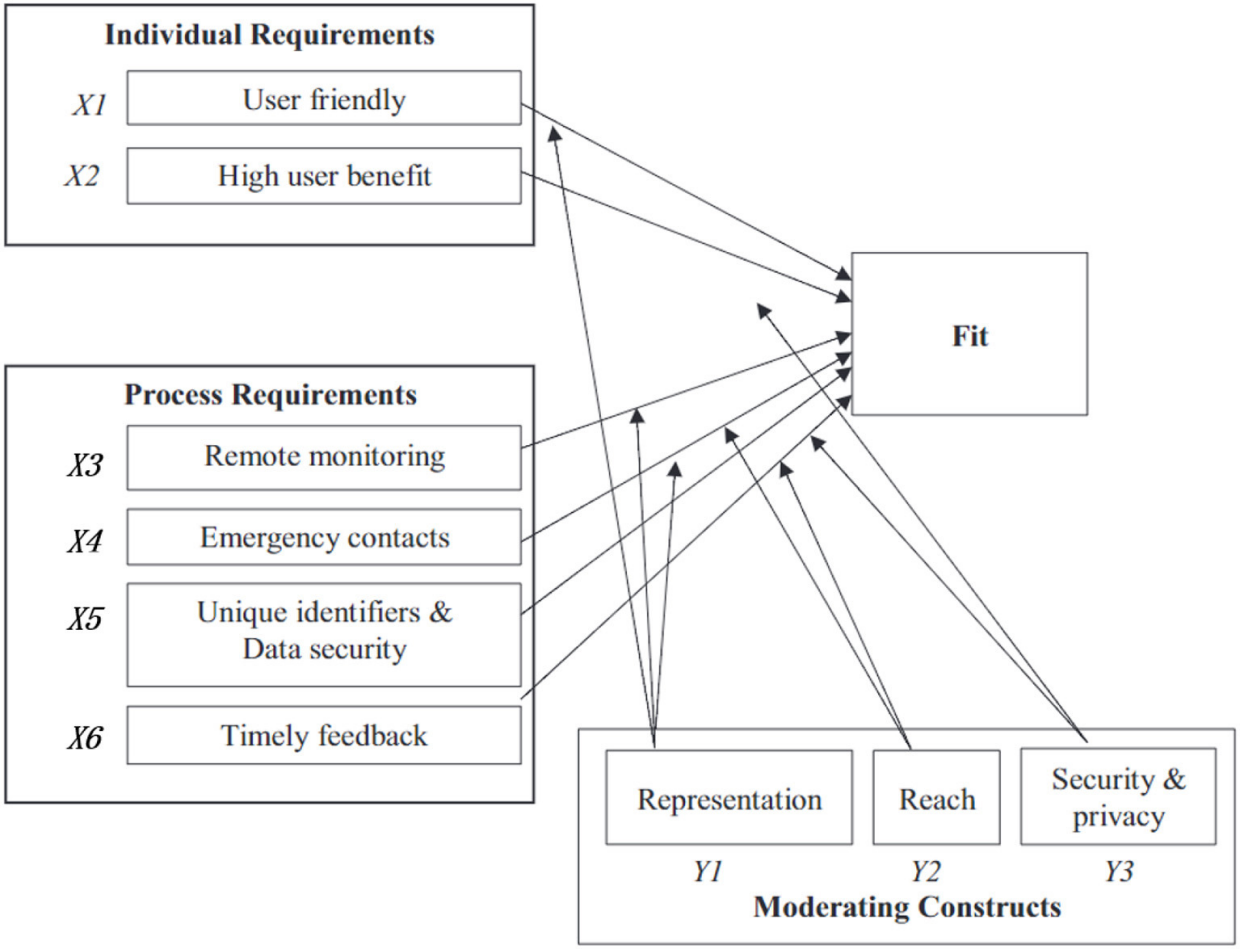
Conceptual framework for designing mHealth solutions.

We adopted an integrating of constructs adapted from the following conceptual models: the Task-Technology Fit (21), the Theory of Planned Behavior (22) and the Process Virtualization Theory (23). A generic schema of various factors are comprised in this conceptual framework. It includes 6 primary constructs (X_1_-X_6_): user friendly, high user benefit, remote monitoring, emergency contacts, unique identifiers And data security, timey feedback and 3 moderating constructs (Y_1_-Y_3_): representation, reach and security and privacy. The primary constructs have negative influence on Fit, while the 3 moderating constructs can moderate the potential negative effect of the 6 primary constructs (Figure 1).

### Study population

Eligibility criteria were an age of more than forty, definite diagnosis of hypertension: Systolic blood pressure (SBP) ≥140 mmHg and/or Diastolic blood pressure (DBP) ≥90 mmHg or being treated with antihypertensive medication, no cognitive deficit and able to possess communication proficiency to carry out study tasks. Participants were excluded if they had cognitive dysfunction, developed serious health conditions that led to hospitalization or death, or had no smart phones to perform the mobile healthcare. Moreover, written informed consent was obtained from all participants during screening.

Study data were collected at baseline and at 12 weeks. The mHealth intervention program included education of healthcare providers, adherence to drug treatment, home-based lifestyle modification, and a mobile health intervention.

### Patient recruitment and randomization

Participants were recruited though the cooperation of local Health and Family Planning Committee (HFPC), which composed of a diverse group of community leaders, township health centers personnel. These cooperative relationships were maintained with regular health advocacy meetings, face-to-face contact, and HFPC events, as described elsewhere (24). Potential participants were directed to local healthcare clinical centers to assess eligibility and to provide informed consent. Eligible participants completed a baseline measurements including BP, waist and hip circumference, height and weight, and a survey consisting of demographics, the Compliance of Hypertensive Patients' Scale (CHPS), self-efficacy, and quality of life. The CHPS is widely used tool for self-reported hypertension compliance scale that was found to be reliable (Cronbach's α = 0.80) (25, 26). This study used the Hypertension Self-efficacy Scale original designed by Han (27) to evaluate the self-efficacy of patients. The test-retest reliability and content validity of the revised version were 0.87 and 0.92, respectively (28). Health-related quality of life was assessed using SF-12 which was a short alternative to the SF-36 (29). The SF-12 has been validated among hypertensive patients and the Cronbach's alpha was 0.801 in our study (30).

Upon receipt of the Bluetooth-enabled BP monitor, potentially eligible individuals were provided with a written instruction manual on how to set up the monitor and properly take a BP measuring. The BP monitor has been approved by BP associations for its accuracy in home use, as described elsewhere (31). Participants were recruited and randomized in a ratio of 1:1 to the control or intervention or using a random number generator. The study staff interacting with patients were not blinded to group assignment, while all the study investigators and data analysts remained blinded until the primary analytic strategies were finalized and all follow-up data were obtained.

### Intervention

The mobile health intervention was the key element, with a complementary text messaging, BP warning, and home-visited intervention. The research team members, who were part of the staff of the local primary care centers, were trained in interactive intervention techniques, performing wearable device, measuring BP, providing life-style modification skills based on the Change Model Stages (32). The motivational training was conducted in a 1-day session, followed with onsite field testing. The research team members visited participants weekly in the first month and every other week thereafter. The mobile health system was developed and formulated though a consensus team including electronics technicians, health care physicians, pharmacists, and patient's family. It included monitoring wearable wristband, mHealth app and website.

All participants were given written information about hypertension and health promotion, and continued to receive routine hypertension management from local clinical centers. Each intervention group participants received a home-based BP monitor wearable wristband that stored and uploaded BP data to a secure website *via* Bluetooth, and then were instructed to transmit at least 1 BP measurements daily. During the first 1 week of the intervention, patients and medical staff of local health centers met everyday *via* telephone until BP measurements data was uploaded and sustained for the whole week, and then the frequency was reduced to weekly.

### Using the model to develop a mHealth intervention

We conducted the mHealth intervention programmes under the guidance of the conceptual framework. Also, we adopted the same conceptual framework to design the intervention strategies which were similar in the constructs but different in detailed content of care needs for the two groups of patients. Table 1 presents examples how we delivered intervention strategies for hypertensive patients based on the conceptual model.

**Table 1:** Use of the conceptual framework to design the mHealth intervention for participants.

**Model element**	**Strategies included in intervention**
**Individual requirements**	
User friendly (*X1*)	We kept the user interface as simple as possible with self-explaining navigation icons. Considering the remoteness of some villages where the internet is unavailable or weak, we made the app operate even without the internet.
High user benefit (*X2*)	The app can send reminder alerts for high BP and due medication to patients. The chat platform in the mHealth app can provide timely support or response from medical staff when patients report any alert signs. These interactive app functions can promote the initiative for app use.
**Process requirements**	
Remote monitoring (*X3*)	Considering the sensory requirements are costly and difficult to virtualize in remote mountains areas, we developed a separate chat platform where multimedia messaging is available.
Emergency contacts (*X4*)	Interaction between health care providers and patients is crucial for health concerns. Virtualization of face-to-face communication *via* video over Internet technology was not feasible in app settings due to high data consumption and limited bandwidth. For any situation that requires emergency medical care, we provided the phone numbers of contracted doctor.
Unique identifiers and Data security (*X5*)	Identification of patients, caregivers and medical care providers is crucial. The patients' mobile phones are the unique identifiers. Health care providers who registered in our mHealth app system have access to all the registered patients. All identifiers and patients' health demographics data security were protected through an encrypted mechanism.
Timely feedback (*X6*)	The patients were provided with website to log health information and free BP monitors which give timely feedback and real-time graphical display about blood pressure fluctuation. Subsequently, health care providers worked with patients to identify health goals and help them link to further health readings available on the website (eg, patient forums, diet advice, videos, and exercise advice).
**Moderating constructs**	
Representation (*Y1*)	Representation refers to the capability of mHealth chat platform to allow communication between patients and medical staff, moderating the potential negative impact of user friendly, remote monitoring and emergency contacts.
Reach (*Y2*)	Reach refers to the capability of mHealth to minimize the medical load and ensure the availability of health care at the fingertips at any time. Consequently, this construct moderate the emergency contacts and timely feedback.
Security and Privacy (*Y3*)	Security and privacy features of mHealth app system can ensure patients' trust in the application. Hence, this construct moderate high user benefit and timely feedback.

### Follow-up assessment

Follow-up assessments were performed at baseline and 12 weeks after enrollment based on intention-to-treat principles for participants. Each assessment included BP measurement using the provided wearable BP monitor, measurement of waist and hip circumference, height and weight, and questionnaire survey.

## Outcomes

The primary outcomes were change in SBP and DBP, and the co-primary outcomes were change in waist and hip circumference, height and weight. The second outcomes were change in self-reported CHPS, self-efficacy, and quality of life.

### Ethical consideration

The study protocol was approved by the ethics committee of School of Health Sciences, Wuhan University in China (Ethical approval number: 2019YF2054). At the process of recruitment, clear explanations about the study objection were provided to all the participants and written informed consent was obtained.

### Statistical analysis

According to the results of our protocol, after the mobile platform management, the BP compliance rate of patients was more than 70%. According to the epidemiological survey conducted by Lin (33) in 125 hospitals in 31 cities in China, the blood pressure compliance rate of outpatients with hypertension was 33.68%, with a conservative estimate of 40%. The parameters and calculation formula of sample size required for the comparison of two sample rates are as follows:


$$\begin{array}{l} n=\left(\frac{\mu_{\alpha }+\mu_{\beta }}{\delta }\right)\;[\pi_{1}\left(1-\pi_{1}\right)+\pi_{2}\left(1-\pi_{2}\right)] \end{array}$$

We sought to recruit at least 134 patients to have 90% power to detect a 5-mm Hg difference in SBP between treatment arms, with an α of 0.05.

We conducted our analyses according to intention-to-treat principles. Means and frequencies of baseline characteristics were calculated between two group differences despite randomization. The primary outcomes and the secondary outcomes were analyzed using univariate linear regression models. We defined statistical significance as *P* < 0.05 and did not adjust our *P-value* threshold for our outcomes, which we assumed would be correlated. In sensitivity analyses, we repeated our analyses for whom the whole complete outcome data were available. Also, we evaluated changes in BP measurements at baseline and the subsequent follow-up assessment using generalized estimating equations with autoregressive errors and an identity link function.

In subgroup analyses, we evaluated differential effects of the intervention on the outcomes with respect to gender, age, number of concomitant diseases, years of hypertension, baseline BMI, baseline hypertension compliance, baseline self-efficacy, and baseline SBP based on the statistical significance of the interaction term for the subgroup of interest in the multivariable model.

All data analyses were conducted using SAS software (version 9.4).

## Results

### Participants

From Nov 2017 to Jul 2018, we screened 200 participants, of whom 148 met eligibility criteria and randomly divided into two equal groups. Eight participants from the intervention group and 6 participants from the control group were lost to follow-up because they could not attend the scheduled meetings despite being contacted by research personnel. Therefore, 66 patients in the intervention group and 68 in the control group completed the final assessment at 12 weeks and were included in the intention-to-treat analysis.

### Baseline characteristics

Demographic and socioeconomic characteristics, systolic blood pressure (SBP), diastolic blood pressure (DBP), BMI, waist circumference (WC), hip circumference (HC), hypertension compliance, self-efficacy, physical health and mental health in the intervention group were similar to those of the control group participants (Table 2).

**Table 2:** Sociodemographic characteristics of participants (*N* = 134).

**Characteristics**	**Intervention**	**Control**	**χ^2^/t**	***P***
	**(*****n** =* **66)**	**(*****n** =* **68)**		
Gender, no. (%)			2.149	0.158
Male	45 (68.18)	38 (55.88)		
Female	21 (31.82)	30 (44.12)		
Age, mean (SD), y	61.37 (11.73)	62.09 (10.66)	0.136	0.713
Ethnic, no. (%)			3.170	0.205
Han	15 (22.73)	25 (36.76)		
Tujia	28 (42.42)	23 (33.83)		
Others	23 (34.85)	20 (29.41)		
Marital status, no. (%)			1.337	0.366
Married	62 (93.94)	60 (88.24)		
Single	4 (6.06)	8 (11.76)		
Years of schooling, *y*			2.080	0.556
≤ 6	17 (25.76)	25 (36.76)		
7–9	10 (15.15)	8 (11.76)		
10–12	15 (22.73)	15 (22.06)		
≥13	24 (36.36)	20 (29.42)		
Years of hypertension, *y*			2.231	0.693
<1	8 (12.12)	11 (16.18)		
1–3	14 (21.21)	13 (19.12)		
3–5	10 (15.15)	7 (10.29)		
5–10	18 (27.27)	15 (22.06)		
>10	16 (24.24)	22 (32.35)		
Number of concomitant diseases, No. (%)			5.294	0.151
0	25 (37.88)	19 (27.94)		
1	20 (30.30)	33 (48.53)		
2	9 (13.64)	9 (13.24)		
≥3	12 (18.18)	7 (10.29)		
SBP, mean (SD), mmHg	152.59 (23.44)	148.85 (20.70)	−0.979	0.329
DBP, mean (SD), mmHg	92.85 (14.93)	91.34 (15.31)	−0.578	0.564
BMI, mean (SD), kg/m^2^	25.55 (2.95)	25.99 (4.20)	0.701	0.485
WC, mean (SD), cm	91.42 (12.92)	90.37 (9.45)	−0.541	0.589
HC, mean (SD), cm	96.74 (8.81)	98.01(6.66)	0.945	0.347
Hypertension compliance, mean (SD)	46.70 (6.69)	46.46 (6.89)	−0.205	0.838
Self-Efficacy, mean (SD)	59.21 (10.44)	57.84 (11.70)	−0.716	0.475
Physical health, mean (SD)	41.68 (9.39)	40.12 (10.30)	−0.912	0.363
Mental health, mean (SD)	48.62 (11.09)	48.72 (9.87)	0.056	0.955

### Blood pressure

At baseline, the mean (SD) SBP was 152.59 (23.44) mmHg in the intervention group and 148.85 (20.70) mmHg among controls. After 12 weeks of follow-up, the mean (SD) SBP decreased by 8.52 (19.73) mmHg in the intervention group and by 1.25 (12.47) mmHg in the control group (between-group difference, −7.265 mm Hg; 95% CI, −12.89 to −1.64 mm Hg; *P* = 0.012) (Table 3). While, there was no difference in the change in DBP between the two groups (between-group difference, −0.41 mm Hg; 95% CI, −3.56 to 2.74 mm Hg; *P* = 0.797).

**Table 3:** Primary and secondary outcomes.

**Variable**	**Intervention group**			**Control group**			**Unadjusted effect estimate**		**Adjusted effect estimate**	
	**Wk 0**	**Wk 12**	**Change**	**Wk 0**	**Wk 12**	**Change**	**Absolute Difference**	***P-value***	**Absolute Difference**	***P-value***
SBP, mmHg, mean (SD)	152.59 (23.44)	144.08 (14.19)	−8.52 (19.73)	148.85 (20.70)	147.6 (17.27)	−1.25 (12.47)	−7.265 (-12.89 to −1.64)	0.012	−0.82 (−4.19 to 2.55)	0.631
DBP, mmHg, mean (SD)	92.85 (14.94)	92.42 (14.12)	−0.42 (10.91)	91.34 (15.31)	91.32 (13.13)	−0.01 (7.19)	−0.41 (-3.56 to 2.74)	0.797	−7.20 (-13.12 to −1.27)	0.018
WC, cm, mean (SD)	91.42 (12.92)	89.29 (12.74)	−2.14 (2.61)	90.37 (9.45)	90.12 (9.34)	−0.25 (0.61)	−1.89 (−2.53 to −1.25)	< 0.01	−1.84 (−2.53 to −1.17)	< 0.001
HC, cm, mean (SD)	96.74 (8.81)	96.44 (8.87)	−0.30 (1.38)	98.01 (6.66)	98.01 (6.65)	−0.01 (0.04)	−0.30 (−0.63 to 0.04)	0.079	−0.32 (−0.67 to 0.03)	0.075
Hypertension compliance, mean (SD)	46.7 (6.69)	54.05 (5.17)	7.35 (7.31)	46.46 (6.89)	49.47 (5.62)	3.01 (4.92)	4.334 (2.210 to 6.46)	< 0.01	3.92 (1.68 to 6.16)	0.001
Self-Efficacy, mean (SD)	59.21 (10.44)	72.11 (4.14)	12.89 (11.95)	57.84 (11.71)	63.26 (9.73)	5.43 (10.54)	7.47 (3.62 to 11.31)	< 0.01	7.89 (3.81 to 11.98)	< 0.001
Physical health, mean (SD)	49.52 (10.10)	61.72 (6.64)	12.21 (10.77)	49.59 (9.11)	51.13 (7.48)	1.54 (7.18)	10.66 (7.54 to 13.78)	< 0.01	10.47 (7.72 to 13.22)	< 0.001
Mental health, mean (SD)	41.68 (9.39)	54.85 (2.04)	13.17 (9.25)	40.12 (10.30)	42.67 (9.19)	2.55 (5.99)	10.62 (7.97 to 13.28)	< 0.01	10.93 (7.74 to 14.12)	< 0.001

Subgroup analyses of the association of the intervention with SBP by gender, age, number of concomitant diseases, years of hypertension, baseline BMI, baseline hypertension compliance and baseline self-efficacy showed no significant between-group differences, while was significant by baseline SBP (*P* < 0.001) (Table 3).

### Waist and hip circumference

At baseline, the mean (SD) WC was 91.42 (12.92) cm in the intervention group and 90.37 (9.45) cm in the control group. After 12 weeks of follow-up, the mean (SD) WC decreased by 2.14 (2.61) cm in the intervention group and by 0.25 (0.61) cm in the control group (between-group difference, −1.89 cm; 95% CI, −2.53 to −1.25 cm; *P* < 0.01) (Table 3). While, there was no difference in the change in HC between the two groups (between-group difference, −0.30 cm; 95% CI, −0.63 to 0.04 cm; *P* = 0.079).

### Hypertension compliance

At baseline, the mean (SD) hypertension compliance was 46.70 (6.69) in the intervention group and 46.46 (6.89) among controls. After 12 weeks of follow-up, the mean (SD) hypertension compliance increased by 7.35 (7.31) in the intervention group and by 3.01 (4.92) in the control group (between-group difference, 4.334; 95% CI, 2.21 to −6.46; *P* < 0.01) (Table 3).

Subgroup analyses of the association of the intervention with hypertension compliance by gender, age, number of concomitant diseases, years of hypertension, baseline BMI, baseline self-efficacy and baseline SBP showed no significant between-group differences, while was significant by baseline hypertension compliance (*P* = 0.003) (Table 4).

**Table 4:** Subgroup analyses of the difference between intervention and control from baseline to 12 weeks.

**Subgroup**	**SBP difference between intervention and control groups (95% CI)**	**Interaction** ***P*-value**	**Hypertension compliance difference between intervention and control groups (95% CI)**	**Interaction** ***P*-value**	**Self-Efficacy difference between intervention and control groups (95% CI)**	**Interaction** ***P*-value**	**Physical health difference between intervention and control groups (95% CI)**	**Interaction** ***P*-value**	**Mental health difference between intervention and control groups (95% CI)**	**Interaction** ***P*-value**
Gender		0.743		0.645		0.478		0.038		0.248
Male	−7.20 (-12.78 to −1.63)		4.43 (2.33 to 6.53)		7.61 (3.80 to 11.42)		10.20 (7.62 to 12.78)		10.46 (7.38 to 13.54)	
Female	−7.53 (-13.81 to −1.24)		3.94 (1.57 to 6.31)		6.89 (2.59 to 11.19)		12.35 (9.45 to 15.26)		11.50 (8.02 to 14.97)	
Age		0.420		0.126		0.903		0.788		0.078
At or below median	−5.75 (-11.38 to −0.13)		4.99 (2.88 to 7.11)		7.84 (3.94 to 11.74)		10.26 (7.57 to 12.95)		11.13 (7.97 to 14.28)	
Above median	-9.44 (-15.25 to -3.63)		3.38 (1.20 to 5.57)		6.93 (2.90 to 10.97)		11.14 (8.36 to 13.92)		9.99(6.73 to 13.26)	
Number of concomitant diseases				0.068		0.097		0.353		
0		0.092								0.774
1	−8.56 (-14.51 to −2.81)		3.56 (1.39 to 5.74)		8.52 (4.53 to 12.51)		10.22 (7.44 to 12.99)		10.67 (7.41 to 13.94)	
2	−7.02 (-13.42 to −0.63)		3.64 (1.27 to 6.01)		7.71 (3.35 to 12.07)		10.65 (7.62 to 13.69)		11.15 (7.58 to 14.72)	
≥ 3	−5.68 (-11.67 to 0.31)		5.46 (3.24 to 7.68)		6.14 (2.06 to 8.68)		11.10 (8.26 to 13.94)		10.50 (7.16 to 13.85)	
Years of hypertension		0.111		0.636		0.921		0.867		0.092
< 1	−1.06 (−8.22 to 6.10)		6.99 (4.30 to 9.67)		11.15 (6.23 to 16.06)		8.58 (5.16 to 12.01)		9.60 (5.55 to 13.66)	
1–3	−7.80 (-13.57 to −2.20)		3.99 (1.83 to 6.16)		6.47 (2.51 to 10.43)		10.73 (7.97 to 13.49)		10.35 (7.08 to 13.62)	
3–5	−7.82 (-13.50 to −2.10)		4.20 (2.07 to 6.33)		7.75 (3.84 to 11.65)		10.87 (8.15 to 13.59)		11.11 (7.89 to 14.33)	
5–10										
>10										
Baseline BMI		0.950		0.475		0.317		0.215		0.163
< 18.5	−15.48 (-47.84 to 16.87)		23.01 (10.93 to 35.08)		51.53 (30.16 to 72.90)		11.79 (-3.79 to 27.31)		22.24 (3.91 to 40.57)	
18.5–24.9	−8.11 (-13.82 to −2.39)		4.47 (2.34 to 6.60)		8.19 (4.41 to 11.96)		11.38 (8.64 to 14.12)		11.21 (7.97 to 14.44)	
≥ 25	−5.58 (-11.28 to 0.11)		4.95 (2.83 to 7.08)		8.41 (4.65 to 12.17)		9.79 (7.06 to 12.52)		10.43 (7.21 to 16.36)	
Baseline Hypertension compliance		0.583		0.003		0.445		0.514		0.905
At or below median	−5.31 (−11.42 to 0.81)		7.30 (5.27 to 9.33)		12.19 (8.38 to 16.00)		9.91 (7.01 to 12.81)		10.43 (7.01 to 13.85)	
Above median	−7.89 (−13.45 to −2.34)		3.38 (1.54 to 5.23)		5.95 (2.49 to 9.41)		10.85 (8.22 to 13.48)		10.74 (7.63 to 13.84)	
Baseline Self-Efficacy		0.328		0.060		< 0.001		0.793		0.926
At or below median	−5.08 (−13.32 to 1.16)		6.84 (4.64 to 9.05)		14.58 (11.11 to 18.06)		10.27 (7.30 to 13.24)		10.81 (7.33 to 14.31)	
Above median	−7.80 (−13.33 to 7.62)		3.73 (1.77 to 5.68)		5.74 (2.66 to 8.83)		10.71 (8.08 to 13.34)		10.62(7.53 to 13.72)	0.416
Baseline SBP		< 0.001		0.684		0.842		0.712		
≤ 160 mmHg	−2.72 (−7.04 to 1.61)		4.54 (2.41 to 6.67)		7.77 (3.91 to 11.63)		10.55 (7.88 to 13.22)		10.80 (7.66 to 13.94)	
>160 mmHg	−17.29 (-21.98 to −12.61)		3.89 (1.58 to 6.20)		6.81 (2.62 to 10.99)		10.79 (7.89 to 13.68)		10.36 (6.96 to 13.76)	

### Self-efficacy

At baseline, the mean (SD) self-efficacy was 59.21 (10.44) in the intervention group and 57.84 (11.71) among controls. After 12 weeks of follow-up, the mean (SD) hypertension compliance increased by 12.89 (11.95) in the intervention group and by 5.43 (10.54) in the control group (between-group difference, 7.47; 95% CI, 3.62 to 11.31; *P* < 0.01) (Table 3).

Subgroup analyses of the association of the intervention with self-efficacy by gender, age, number of concomitant diseases, years of hypertension, baseline BMI, baseline hypertension compliance and baseline SBP showed no significant between-group differences, while was significant by baseline self-efficacy (*P* < 0.001) (Table 4).

### Quality of life

At baseline, the mean (SD) physical health was 49.52 (10.10) in the intervention group and 49.59 (9.11) in the control group, and the mean (SD) mental health was 41.68 (9.39) in the intervention group and 40.12 (10.30) in the control group. After 12 weeks of follow-up, the mean (SD) physical health increased by 12.21 (10.77) in the intervention group and by 1.54 (7.18) in the control group (between-group difference, 10.66; 95% CI, 7.54 to 13.78; *P* < 0.01), the mean (SD) mental health increased by 13.17 (9.25) in the intervention group and by 2.55 (5.99) in the control group (between-group difference, 10.93; 95% CI, 7.74–14.12; *P* < 0.01) (Table 3).

Subgroup analyses of the association of the intervention with mental health by age, number of concomitant diseases, years of hypertension, baseline BMI, baseline hypertension compliance, baseline self-efficacy, and baseline SBP showed no significant between-group differences, while was significant by gender (*P* = 0.038) (Table 4). While, Subgroup analyses of the association of the intervention with mental health by all of them showed no significant between-group differences (Table 4).

## Discussion

To our knowledge, this study is the first randomized controlled trial to assess mHealth intervention to improve cardiovascular factors and promote healthier lifestyle behaviors among individuals at high risk of cardiovascular disease in low-resource rural settings in China. This study aimed to evaluate the effects of mobile phone-based intervention on BP control, waist and hip circumference, self-reported hypertension compliance, self-efficacy, and quality of life. Our findings show that compared with local usual primary care, mHealth BP monitoring intervention resulted in significant improvements in SBP and other cardiovascular factors. Compared with usual community-based management of hypertension patients, mHealth intervention patients had greater controlled SBP, waist and hip circumference. Moreover, the intervention also improved some aspects of self-reported hypertension compliance and self-efficacy, and appeared to have an acceptable level of quality of life.

The results of this randomized control trial showed that the wearable BP wristband and app-based management could decreased SBP by 8.52 (19.73) mm Hg (95% CI, −12.89 to −1.64 mm Hg; *P* = 0.012), which showed similar treatment effects of medication treatment. A recent meta-analysis (34) that analyzed 14 RCTs showed that intensive BP-lowering medication treatment could decrease SBP by an additional 8.3 mmHg (95% CI: 2.1–14.1 mmHg), which could resulted in 14% reduction of cardiovascular disease (CVD) risk. In line with our findings, a RCT on 1,372 hypertension patients reported that mobile phone text messages could resulted in a small reduction in SBP compared with usual care after 12 months intervention (6). Also, it was reported that observations, including in-person visits, telephone support, and text messaging may have important implications when conducting internet-based interventions (35, 36). So we added mobile devices, including phone calls, short message service, face-to-face communication *via* video and in-person visits as our intervention methods. However, according to our literature review, there is no clear explanation for the different intervention results of the SBP and DBP.

Unique features of our study were the significant improvement in self-reported hypertension compliance and self-efficacy with corresponding reductions in SBP. In our study, readings from the home-based BP monitoring wearable devices were used to evaluate trial outcomes. A possible explanation might be that the reductions in BP from baseline to the 12 weeks of follow-up that we observed in both the control and intervention group were resulted from fluctuations in these home BP monitoring readings, and that the magnitude of these fluctuations was larger than the hypothesized effect from the smartphone application (37). Hence, all participants were engaged in some level of self-monitoring. In this respect, the home-based BP monitoring intervention have significant positive effects on BP control (38), hypertension compliance (6) and self-efficacy (39) and may have been particularly motivating for the patients in our trial.

It is interesting to note a net reduction in the waist circumference, while, no changes were seen in levels of hip circumference. It might be related to the amount of exposure to the intervention domains defined by the patients during motivational suggestion, following the autonomy support on the basis of principle. Thus, target behavior including reduction of high-sugar and high-fat foods intake was most commonly chosen during motivational home visit or counseling calls. In line with our findings, Partridge et al. (40) conducted a 12-week mHealth prevention program, with weekly goal setting to prevent weight gain and improve lifestyle behaviors among overweight young adults.

How could home-based BP monitoring wearable devices enhance the quality of life for patients with hypertension? While the wearable devices we tested has received high usability scores? It may be the reason that patients with hypertension in low-resource rural settings have needs that differ from those with other conditions (20, 24). Therefore, smart tools shown greater effects on clinical outcomes when they linked with additional support, especially though connection to health care professionals (41). Meanwhile, it seems the individuals would be highly adherent to their hypertension compliance to derive clinical benefits (42). If the highly adherent from the intervention could persist more than 12-week duration of our trial, it may be possible that we could have observe more significant life quality improvements with longer follow-up. Finally, quality of life was measured by self-report. Although, the SF-12 questionnaires has been validated and extensively used, self-reported tools are difficult to avoid social desirability bias and may overestimate true condition (43). As such, after exposure to a home-based BP monitoring device that very clearly encouraged adherence, intervention group participants may have been more likely to report higher level life quality without actually changing their physical or mental health condition.

Several limitations should be considered of this trial. The sample size was small and included only 6 primary care centers, and excluded those had no smartphones, which may contributed discrepancies in participant baseline characteristics and lack of power to detect differences of the secondary and subgroup analyses outcomes between two groups. Also, the hypertension compliance, self-efficacy and SF-12 questionnaires were all self-reported measurements, therefore we cannot conclude the findings to a broader population. In addition, the trial was not double-blinded, which may lead to an effect on the reporting bias such as recall error, social desirability or other subjective outcomes. However, BP recordings were measured by automated wearable devices with a standard protocol, which was unlikely to have been biased. Lack of information on long-term intervention effects, reimbursement mechanisms, and return on investment have been revealed as barriers to trail implementation (44). Future studies should be conducted to address these issues when a planned long-term follow-up study.

## Conclusions

Despite the popularity of smartphone health-related apps has increased quickly, there has been a lack of rigorous studies which including a clinically important outcome (45, 46). Our trial, to our knowledge, is one of the first randomized clinical studies using a conceptual framework (47), reporting the effect of a stand-alone mHealth platform to improve DBP control and increase hypertension compliance, self-efficacy and life quality. We found mHealth platform was safe and effective for promoting hypertension compliance, self-efficacy, life quality and DBP control, but no difference in SBP between the control and intervention groups during 12 weeks. If these finding are found to be stable and cost-effective during an even longer intervention period, it should spur wider testing and dissemination of similar alternative platform to manage hypertension and other chronic conditions.

## Data availability statement

The original contributions presented in the study are included in the article/supplementary material, further inquiries can be directed to the corresponding author.

## Ethics statement

The studies involving human participants were reviewed and approved by the Ethics Committee of the School of Public Health, Wuhan University. The patients/participants provided their written informed consent to participate in this study.

## Author contributions

ZY conceived the study and completed the original draft preparation. ZY and TX collected data. TX provided the recruitment resources. WQ reviewed, edited the final draft, and received the funding. All authors contributed to the article and approved the submitted version.
